# Mitigating the impact of climate change on plant productivity and ecosystem sustainability

**DOI:** 10.1093/jxb/erz518

**Published:** 2020-01-07

**Authors:** Ashwani Pareek, Om Parkash Dhankher, Christine H Foyer

**Affiliations:** 1 Stress Physiology and Molecular Biology Laboratory, School of Life Sciences, Jawaharlal Nehru University, New Delhi, India; 2 Stockbridge School of Agriculture, University of Massachusetts, Amherst, MA, USA; 3 School of Biosciences, College of Life and Environmental Sciences, University of Birmingham, Edgbaston, UK

**Keywords:** Climate change, drought, food security, heat stress, rice, salinity, wheat

**Climate change exerts adverse effects on crop production. Plant researchers have therefore focused on the identification of solutions that minimize the negative impacts of climate change on crops. This Special Issue comprises 23 articles that celebrate the 2020 International Year of Plant Health, highlighting the processes, mechanisms, and traits that will underpin future sustainability of crop quality and yield. These articles critically evaluate recent advances in our understanding of climate change impacts on plants, within the context of climate-smart agriculture.**

## Background concepts and considerations

Global agriculture is dependent on a relatively small number of crop species, which have been bred to optimize productivity within a relatively narrow range of environmental variations (Kukal and Irmak, 2018). Moreover, current food security has been achieved through intensive industrial agriculture, in which large farms often grow the same crops annually, using large amounts of pesticides and fertilizers that ultimately deplete soils, pollute water, cause nutrient loss, decrease biodiversity, and contribute to climate change. Most developed countries have had to embrace modern agricultural technologies in order to achieve food security for increasing populations, as well as to support agri-business and income generation. Although, the ecological consequences of technocentric approaches to food production have been appreciated for many decades, it is only relatively recently that the negative environmental impact of agriculture has come into sharp focus. Agricultural emissions (carbon dioxide, methane, and nitrous oxides) particularly linked to livestock production systems amount to about a quarter of global greenhouse gas emissions. The significant climate footprint of food production is similar to that of burning fossil fuels. There is growing agreement that current agricultural practices are not sustainable, because they tend to squander valuable resources and degrade the environment. These considerations have changed the ethos of basic plant science research and direction of demand-led plant breeding to focus more on mechanisms and processes that allow plants to be healthy and grow well on limiting resources. Next-generation crop plants need to be water and nutrient use efficient, and have sustainable yields over a wider range of environmental conditions.

On top of the uncertainty regarding the future environmental impact of agriculture comes the looming threat to yield sustainability caused by climate change-induced fluctuations in weather patterns (Porfirio *et al.*, 2018). Predictions suggest that on a global scale, an increase in land use of ~100 Mha with a tripling of international trade is required by 2050 to meet the future crop demands of 9.8 billion people, without causing any significant change in existing cropped land area (Pastor *et al.*, 2019). Extreme weather events cause enormous damage to crop production. Mitigation strategies to combat the effects of such extreme events, which are destined to become much more frequent, are required alongside the global drivers of agricultural production (Dhankher and Foyer, 2018; Schewe *et al.*, 2019; Nutan *et al.*, 2020a). This Special Issue focuses on the ways in which plant science is poised to meet these challenges and mitigate the impact of climate change in accordance with the United Nations Sustainable Development Goals. To celebrate the 2020 International Year of Plant Health, the contents of this Special Issue consider how plant biology may be tailored to meet the challenges posed by climate change. Together, these papers present and discuss the impacts of environmental stresses on different crops, providing authoritative insights into the mechanisms, processes, and genes/gene networks that will allow mitigation of negative effects. We highlight how increases in current knowledge will provide effective solutions and drive strategies for future plant improvement and breeding.

The concept that recent developments in plant science have the potential to address the challenges facing agriculture and food production is widely accepted (Bailey-Serres *et al.*, 2019). There are now a range of strategies available for enhancing sustainable crop production and resilience to climate change including high-throughput single nucleotide polymorphism (SNP) genotyping, genomic selection and trait mapping. These tools are essential not only for an in-depth understanding of trait variations but also for the transformative engineering required to accelerate plant breeding efforts. The ‘pyramid’ approach to introducing favourable alleles and genes combinations is discussed in the review by Nutan *et al.* (2020a), which summarizes the physiological and molecular architecture that determines rice yield traits under various environmental (drought, salinity) stresses. This authoritative treatise suggests various gene combinations that may be used to improve rice grain yields under optimal and stress conditions. The importance of the accurate selection of key candidate structural and regulatory genes underpinning selected traits for applications using gene editing tools is discussed in the paper by Zafar *et al.* (2020). These authors present the opinion that the CRISPR/Cas9 system provides an efficient and practical solution to the production of improved crop varieties with a greater sustainability of yield and hence better resilience to climate change. However, much depends on producer adoption and favourable economic, policy and other framework conditions to actually realize any of the pathways and their benefits to crop production.

## Water use efficiency and drought tolerance

**Fig. 1.**
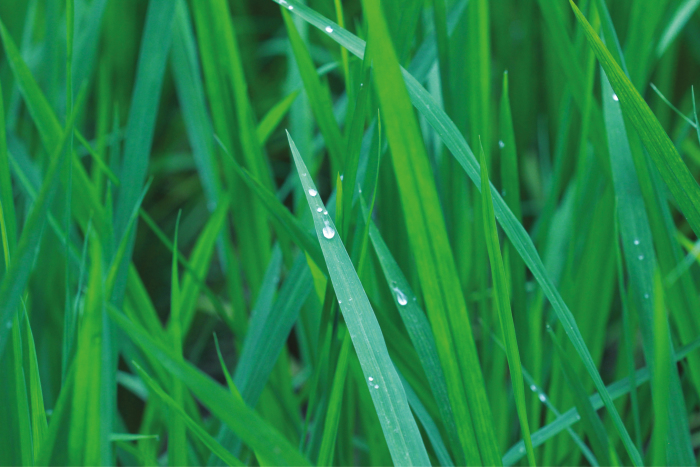
Rice growing in the field. Rice cultivation requires large amounts of water. (Photo courtesy of Rohit Joshi and Ashwani Pareek, India.)

Water deficits pose a serious threat to crop productivity and food security in many parts of the world due to poor or erratic rainfall and depletion of groundwater reserves (Hussain *et al.*, 2019). Improvements in crop productivity under conditions of limited water availability are vital to meet global food demand (Balyan *et al.*, 2017). Agricultural crop production requires substantial amounts of water. For example, it has been calculated that 2497 litres of water are required to produce 1 kg of rice (Fig. 1; Rahaman *et al.*, 2016). Therefore, the development of improved rice genotypes with increased water use efficiency is essential without compromising yields (Shahane *et al.*, 2019). Climate change is predicted to increase the frequencies of droughts and floods, both of which will be problematic for food production (Mar *et al.*, 2018). In China, variations in rainfall have already led to a water crisis and a severe decline in rice production (Yao *et al.*, 2017). Recent strategies such as growth enhancements or increases in photosynthetic efficiency have the potential to increase intrinsic yields (Ambavaram *et al.*, 2014). The identification of new molecular markers and their effective utilization in plant breeding will accelerate the production of improved crop cultivars that are more tolerant to drought and other stresses.

Several manuscripts in this issue (Kunert and Vorster, 2020, Melandri *et al.*, 2020a; Nutan *et al.*, 2020*b*; Sulpice, 2020; Ye *et al.*, 2020) highlight the physiological, molecular, and biochemical responses of plants to drought stress. A metabolite profiling analysis of the flag leaves of 292 *indica* rice accessions has led to the identification of new molecular markers for drought tolerance and sensitivity in terms of grain yield (Melandri *et al.*, 2020a; Sulpice, 2020). The article by Melandri *et al.* (2020a) highlights the central role of the ascorbate–glutathione cycle and of lipid peroxidation in mitigating drought-induced yield losses. Dehydroascorbate reductase activity and malondialdehyde levels were shown to be accurate biomarkers for drought tolerance. These markers have potential use in breeding for improved rice grain yield stability under drought. An association mapping and genetic dissection study of drought-induced canopy temperature differences in rice is reported in another paper by Melandri *et al.* (2020*b*). Intriguingly, these authors report that low canopy temperature is a useful indicator of access to moisture during drought (Kaler *et al.*, 2018). Ye *et al.* (2020) describe an important strategy for soybean yield improvement. These authors have identified quantitative trait loci (QTLs) regulated by slow canopy wilting (SW) in late maturing soybean genotypes (Ye *et al.*, 2020). The SW trait, which is associated with drought tolerance, involves at least two distinct mechanisms: water use efficiency and conservation. Since drought already causes ~40% reduction in soybean yields (Specht *et al.*, 1999), the findings reported by Ye *et al.* (2020) represent an important new direction of research (Kunert and Vorster, 2020). These authors have also identified genetic resources for improving drought tolerance in early maturity group soybeans.

## Salinity stress

**Fig. 2.**
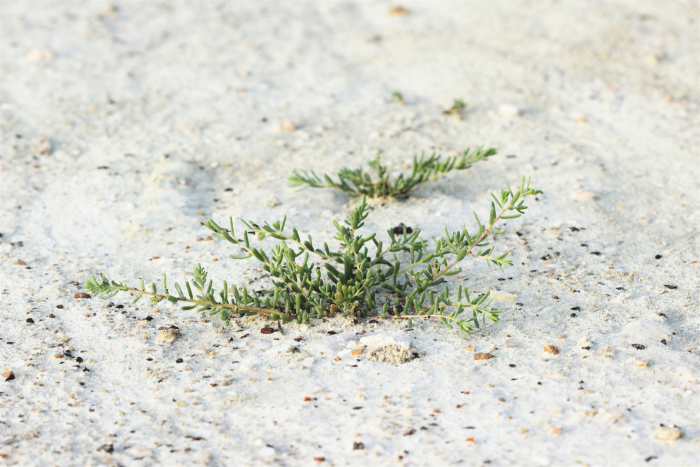
*Suaeda fruticosa* growing on saline sandy soil. (Photo courtesy of Wungrampha Silas and Ashwani Pareek, India.)

Salinity stress is an important yield-limiting factor that poses a significant threat to agriculture worldwide. The identification of traits that underpin salt stress tolerance is the prerequisite to develop improved cultivars (Akrami and Arzani, 2019). This requires a better understanding of stress tolerance mechanisms, for example in halophytic species such as *Suaeda fruticosa* (Fig. 2), which can be used as a model system for studies on salinity tolerance (Flowers and Colmer, 2008). *Suaeda fruticosa* can not only survive but also complete its life cycle in conditions of soil salinity of 65 dS m^–1^, pH of 10.5, and under little or no water (Wungrampha *et al.*, 2019). Several papers in this issue focus on improving salinity tolerance in crop plants (Hartley *et al.*, 2020; Joshi *et al.*, 2020; Nongpiur *et al.*, 2020; Nutan *et al.*, 2020*b*). Potential mechanisms for osmosensing in plants are expertly discussed by Nongpiur *et al.* (2020), who highlight the roles of key proteins such as receptor-like kinases, mechanosensitive calcium channels, phospholipase C, aquaporins, and membrane-bound histidine kinases as osmosensors in stress perception. These osmosensors, which may serve as master regulators of the osmotic stress response, are useful targets for the development of osmotic stress-tolerant crops.

Understanding genetic variations in plant responses to salinity stress and associated traits is critical for improving plant adaptation to saline conditions. Hartley *et al.* (2020) report the findings of a genome-wide association study (GWAS) on diverse rice genotypes that has led to the identification of three QTLs associated with potassium use efficiency (KUE). The requirement for potassium in multiple plant processes and hence its central importance in attaining high crop yields and ecosystem stability is expertly discussed in the review by Srivastava et al., (2020). In their comprehensive consideration of this topic, these authors discuss the complexity of the signalling network that involves reactive oxygen species (ROS), calcium and phytohormones for the sensing of potassium deficiency in plants. Since relatively little soil potassium is available in forms that are accessible to plants, the authors consider the possibilities for the successful application of genetic approaches using potassium transporters to increase plant KUE and so achieve sustainable food production. Like potassium, sodium is an essential plant nutrient. It is not surprising therefore that a sodium transporter gene called OsHKT2;1 was shown to be a key player in sodium/potassium interactions underpinning KUE (Hartley *et al.*, 2020). Furthermore, overexpression of OsGATA8 localized in the *Saltol* QTL region in Arabidopsis and rice imparted tolerance to salinity and drought, together with improved yield (Nutan *et al.*, 2020*b*). Moreover, this study provides evidence that OsGATA8 regulates the expression of critical genes involved in stress tolerance, including those encoding ROS scavenging and chlorophyll biosynthesis proteins. A further important study reported in this issue characterized marker-free transgenic rice lines overproducing trehalose. This was achieved by overexpression of genes associated with trehalose-6-phosphate synthase/phosphatase-mediated regulation of sugar metabolism. Remarkably, this metabolic engineering led to improved yield potential even under saline–alkaline conditions (Joshi *et al.*, 2020). A metabolic profiling of resulting rice lines showed that overproduction of trehalose in leaves differently modulates other metabolic switches leading to significant changes in the levels of sugars, amino acids, and organic acids in transgenic plants under control and stress conditions.

## High temperature stress

Climate change-led increases in local and global temperatures pose a significant threat to plant growth and crop production (Priya *et al.*, 2019). The Intergovernmental Panel on Climate Change reported that if current rates of global warming continue, global temperatures will continue to increase by a further 1.5 °C between 2030 and 2052 (Intergovernmental Panel on Climate Change, 2018). Heat stress can impair all stages of plant growth from germination to reproduction, limiting the productivity of major staple food crops (Hussain *et al.*, 2019). For example, heat stress has a negative impact on wheat yields. A 4–6% reduction in average global yields of wheat is predicted for each 1 °C increase in global mean temperature (Asseng *et al.*, 2015). Current concepts concerning heat stress effects on source–sink relationships and metabolome dynamics in wheat is competently reviewed by Abdelrahman *et al.* (2020), who place emphasis on the temperature susceptibility of the reproductive and grain-filling stages, and discuss the selection and development of germplasm that can maintain high yields under heat stress. The plant reproductive organs and processes leading to seed set are extremely vulnerable to increasing temperatures. Our current knowledge and understanding of the molecular mechanisms that contribute to this temperature sensitivity are ably discussed by Lohani *et al.* (2020), who summarize the regulation of male and female reproductive organ development and fertilization, together with heat-induced abnormalities at flowering. This review highlights the high-temperature-sensitive stage-specific bottlenecks in sexual reproduction.

The importance of genetic mechanisms in the heat stress responses of crop plants is described in the review by Janni *et al.* (2020), who evaluate the potential roles of different processes in increasing crop resilience and productivity. A metabolite profiling analysis of winter wheat genotypes revealed a significant increase in sugars, sugar‐alcohols, and phosphate in the more temperature-tolerant genotypes (Impa *et al.*, 2019). Carbon loss caused by high night‐time temperatures led to a significant reduction in winter wheat yields (Impa *et al.*, 2019). The study by Sharma *et al.* (2020) demonstrates that plant growth regulators (PGRs) can afford protection against high-temperature stress (HTS). These authors report that PGR-treated plants were more resilient to heat stress in terms of less damage to membranes, improved photosynthesis and leaf water status, and carbon allocation than the untreated HTS controls (Sharma *et al.*, 2020).

## Soil health

Soil health and fertility are not only important to sustainable agriculture but they are also key considerations in poverty alleviation and the improvement of livelihoods of resource-poor farmers (Heger *et al.*, 2018). Several manuscripts in this issue focus on different aspects of climatic change impacts on soil fertility (Jiang *et al.*, 2020; Middendorf *et al.*, 2020; Stewart *et al.*, 2020). The review by Stewart *et al.* (2020) provides a comprehensive survey of the barriers to crop productivity and improving soil fertility in sub-Saharan Africa, providing evidence-based recommendations. The holistic solutions described by Stewart *et al.* (2020) cover socio-economic considerations, farming system approaches, and soil management strategies using inorganic and organic sources of nutrients, leading to highly recommended solutions to current soil fertility issues in sub-Saharan Africa. The evidence-based process and methodology for prioritizing recommendations will be extremely useful for future action plans, investments, and strategies deployed in sub-Saharan Africa as well as other parts of the developing world. The review by Middendorf *et al.* (2020) describes climate change effects on global crop productivity, stressing the need for an interdisciplinary and multinational initiative to develop better models for determining research priorities for climate-resilient agriculture. These authors highlight the importance of participatory approaches that provide a variety of perspectives in order to gain insights into critical issues such as defining and understanding sustainable intensification, climate-smart agriculture, and soil fertility prioritization in sub-Saharan Africa.

Endophytes and microbial symbionts such as bacteria, fungi, or yeast can provide substantial benefits for plant growth and development, particularly under conditions of environmental stress. Symbiotic endophytes can also facilitate better CO_2_ diffusion in rice leaves grown under elevated atmospheric CO_2_ conditions. This strategy alleviated the drought stress-induced inhibition of photosynthesis and improved water use efficiency in rice (Rho *et al.*, 2020).

Biochars are carbon-rich materials that can be added to soils to greatly enhance the moisture content (Burrell *et al.*, 2016), soil organic carbon content (Luo *et al.*, 2016), and nutrient retention capacity (Peng *et al.*, 2011) to increase sustainable crop production. The review by Jiang *et al.* (2020) summarizes the biochar modification approaches (physical, chemical, and biochar-based organic composites) to soil remediation and discusses the potential role of biochar in sustainable crop production and soil resilience, including the degradation of soil organic matter, the improvement of soil quality, and reductions in greenhouse gas emissions.

## Nanotechnology and plant health

Phytopathogens cause estimated crop losses of up to 20–30% annually (Kashyap *et al.*, 2017). Changing climatic conditions, particularly global warming, are considered to favour pathogen expansion and increase the aggressiveness of infestation (Bebber *et al.*, 2013). Moreover, some authors consider that elevated atmospheric CO_2_ levels may increase the susceptibility of host plants (Juroszek and von Tiedemann, 2011). The global use and demand of pesticides and synthetic toxic agrochemicals may therefore increase (FAOSTAT, 2019). The use of engineered nanomaterials (NMs) in agriculture is rapidly increasing, with applications ranging from nanofertilizers to nanopesticide/insecticides. NMs, due to their unique properties, offer a promising alternative as a less toxic and sustainable product in plant disease management (Elmer and White, 2018). The review by Fu *et al.* (2020) describes the potential applications of NMs in crop disease management, together with the benefits for crop adaptation measures.

## Oxidative stress and cellular antioxidants

Respiratory pathways are crucially important determinants of plant defences against abiotic stresses and climate change. The importance of the alternative oxidase (AOX) in preventing the respiratory production of ROS and reactive nitrogen species (RNS) is discussed in the review by Florez-Sarasa *et al.* (2020). These authors consider how the AOX pathway enables plants to deal with gaseous pollutants such as elevated carbon dioxide (CO_2_), nitrogen oxides (NOx), and ozone (O_3_).

Glutathione (GSH) is the key redox molecule in all living cells and is directly involved in the maintenance of intracellular redox homeostasis. GSH is involved in numerous cellular processes such as detoxification of heavy metals and metalloids (Dhankher *et al.*, 2002; Paulose *et al.*, 2013). The study described by García-Quirós *et al.* (2020) highlights the importance of the GSH pool in the development of reproductive tissues and in pollen tube growth in *Arabidopsis thaliana.* Reporter lines expressing the redox-sensitive green fluorescent protein (roGFP2) were used to measure the glutathione redox state of cells in each part of the flower. This study reveals that the ungerminated pollen resides in a highly oxidized state that is commensurate with quiescence. Moreover, analysis of the glutathione-deficient *cad2-1* (cad2-1/roGFP2) mutants revealed that the pollen achieves high GSH/GSSG ratios upon germination and that this highly reduced state is required to sustain pollen tube growth (García-Quirós *et al.*, 2020).

The compartmentation of proteins is a fundamental concept in cellular regulation, but it has long been known that the location of many proteins is not totally fixed and that some proteins can relocate between different intracellular compartments, particularly between the cytosol and nucleus. The proteins and processes involved in redox-dependent intercompartmental switching is expertly discussed in the review by Foyer *et al.* (2020). These authors provide an insightful overview of the topic that highlights new advances in our current understanding of protein movement and relocation, considering that redox-dependent processes could underpin protein relocation between different cellular compartments in response to metabolic or environmental triggers. These authors describe how redox post-translational modifications (PTMs) can control the compartmentation of many proteins, including antioxidant and/or redox-associated enzymes.

Finally, our current knowledge of the impacts of climate and related stresses on physiological processes in woody tree species and vine crops remains poor. The study reported by Signorelli *et al.* (2020), which uses a combination of micro-computed tomography, histology, and oxygen microsensors to study grapevine buds is an elegant example of contemporary physiological approaches to tackle this problem. Signorelli *et al.* (2020) demonstrate that the apoplastic pore size of the grapevine latent bud is highly regulated, and that this probably forms an important feature of the seasonal behaviour and resilience of the species.
